# Clinical pharmacokinetics of 3-h extended infusion of meropenem in adult patients with severe sepsis and septic shock: implications for empirical therapy against Gram-negative bacteria

**DOI:** 10.1186/s13613-019-0622-8

**Published:** 2020-01-10

**Authors:** Amol T. Kothekar, Jigeeshu Vasishtha Divatia, Sheila Nainan Myatra, Anand Patil, Manjunath Nookala Krishnamurthy, Harish Mallapura Maheshwarappa, Suhail Sarwar Siddiqui, Murari Gurjar, Sanjay Biswas, Vikram Gota

**Affiliations:** 1 Department of Anesthesiology, Critical Care and Pain, Tata Memorial Centre, Homi Bhabha National Institute, Mumbai, India; 2 Department of Anesthesiology, Critical Care and Pain, Tata Memorial Hospital, Homi Bhabha National Institute, Mumbai, India; 3 Department of Clinical Pharmacology, Advanced Centre for Treatment, Research and Education in Cancer (ACTREC), Tata Memorial Centre, Homi Bhabha National Institute, Kharghar, Navi Mumbai, Mumbai, India; 4 Department of Critical Care Medicine, Mazumdar Shaw Medical Centre, Narayana Health, Bengalore, India; 5 Department of Critical Care Medicine, King George’s Medical University, Lucknow, Uttar Pradesh, India; 6 Department of Microbiology, Tata Memorial Hospital, Homi Bhabha National Institute, Mumbai, India

**Keywords:** Meropenem dosing, Anti-bacterial agents, Antimicrobial pharmacokinetics, Septic shock

## Abstract

**Background:**

Optimal anti-bacterial activity of meropenem requires maintenance of its plasma concentration (Cp) above the minimum inhibitory concentration (MIC) of the pathogen for at least 40% of the dosing interval (fT > MIC > 40). We aimed to determine whether a 3-h extended infusion (EI) of meropenem achieves fT > MIC > 40 on the first and third days of therapy in patients with severe sepsis or septic shock. We also simulated the performance of the EI with respect to other pharmacokinetic (PK) targets such as fT > 4 × MIC > 40, fT > MIC = 100, and fT > 4 × MIC = 100.

**Methods:**

Arterial blood samples of 25 adults with severe sepsis or septic shock receiving meropenem 1000 mg as a 3-h EI eight hourly (Q8H) were obtained at various intervals during and after the first and seventh doses. Plasma meropenem concentrations were determined using a reverse-phase high-performance liquid chromatography assay, followed by modeling and simulation of PK data. European Committee on Antimicrobial Susceptibility Testing (EUCAST) definitions of MIC breakpoints for sensitive and resistant Gram-negative bacteria were used.

**Results:**

A 3-h EI of meropenem 1000 mg Q8H achieved fT > 2 µg/mL > 40 on the first and third days, providing activity against sensitive strains of Enterobacteriaceae, *Pseudomonas aeruginosa* and *Acinetobacter baumannii*. However, it failed to achieve fT > 4 µg/mL > 40 to provide activity against strains susceptible to increased exposure in 33.3 and 39.1% patients on the first and the third days, respectively. Modeling and simulation showed that a bolus dose of 500 mg followed by 3-h EI of meropenem 1500 mg Q8H will achieve this target. A bolus of 500 mg followed by an infusion of 2000 mg would be required to achieve fT > 8 µg > 40. Targets of fT > 4 µg/mL = 100 and fT > 8 µg/mL = 100 may be achievable in two-thirds of patients by increasing the frequency of dosing to six hourly (Q6H).

**Conclusions:**

In patients with severe sepsis or septic shock, EI of 1000 mg of meropenem over 3 h administered Q8H is inadequate to provide activity (fT > 4 µg/mL > 40) against strains susceptible to increased exposure, which requires a bolus of 500 mg followed by EI of 1500 mg Q8H. While fT > 8 µg/mL > 40 require escalation of EI dose, fT > 4 µg/mL = 100 and fT > 8 µg/mL = 100 require escalation of both EI dose and frequency.

## Background

Meropenem is a broad-spectrum injectable carbapenem commonly used for empirical therapy of severe sepsis or septic shock. Anti-bacterial activity of meropenem is related to the fraction of time (fT) between doses during which the plasma concentration (Cp) is maintained above the minimum inhibitory concentration (MIC) for the infecting organism [1]. In vitro and in vivo animal models suggest that for the optimal bactericidal activity for carbapenems, the Cp must remain above the MIC for the pathogen for at least 40% of dosing interval (fT > MIC > 40) [2, 3]. Other pharmacokinetic (PK) targets like targeting Cp more than four times of MIC for at least 40% of dosing interval (fT > 4 × MIC > 40) and continuous exposure of meropenem above the MIC (fT > MIC = 100 and fT > 4 × MIC = 100) have also been suggested. The fT > MIC can be increased by prolonging the duration of infusion for β-lactams [4]. Extended infusions (EI), with the dose delivered over several hours, and continuous infusions over 24 h have been proposed in place of the usual intermittent infusions given over a few minutes to an hour, to improve the pharmacokinetic/pharmacodynamic (PK/PD) properties [5–13].

Delay in the administration of effective antibiotics is associated with a measurable increase in mortality in patients with septic shock [14, 15]. The Surviving Sepsis Campaign guidelines 2012 recommend effective antibiotics within an hour of diagnosis of severe sepsis or septic shock [16]. Logically, antibiotics would be effective only when the Cp crosses the MIC of the organism; hence it may be prudent to achieve Cp > MIC as early as possible.

In patients with severe sepsis or septic shock, meropenem pharmacokinetics are altered due to a variety of reasons. These include increased volume of distribution due to fluid loading and altered vascular permeability, augmented renal clearance mainly due to increased cardiac output, or impaired renal clearance due to renal dysfunction [5, 17, 18].

This study was planned to evaluate the pharmacokinetics of the existing practice of 3-h EI of 1000 mg meropenem without a preceding bolus in patients with severe sepsis or septic shock and its implications for optimal dosing of the drug in this setting. The primary objective was to determine the proportion of patients achieving fT > MIC > 40 for sensitive strains of Enterobacteriaceae and *Pseudomonas aeruginosa* (PsA) and *Acinetobacter baumannii* (AcB) with MIC breakpoints 2 µg/mL [19].We also planned to look at the proportion of patients in whom Cp > MIC would be achieved within 1 h of starting the first infusion. Samples collection both on day 1 and day 3 was planned to look at the changes in PK parameters over a period of time. Modeling and simulation were planned to simulate other targets such as fT > 4 × MIC > 40, fT > MIC = 100, and fT > 4 × MIC = 100.

## Materials and methods

### Study design

Prospective observational study.

### Setting

This study was conducted in a 14-bed medical–surgical intensive care unit (ICU) in a university-affiliated cancer center. The study was approved by the institutional review board. Patients received meropenem as a standard of care as decided by the treating intensivist. Written informed consent was obtained from the patients or their legal representatives at the time of ICU admission for blood sample collection for meropenem assay.

### Participants

We included patients aged 18–70 years of either sex, with known or suspected severe sepsis or septic shock admitted to the ICU and receiving meropenem 1000 mg 3-h extended infusion (EL) eight hourly (Q8H) as a standard of care. Patients who had received any carbapenem in the previous 72 h, those with baseline predicted creatinine clearance less than 50 mL/min [20] and those not expected to survive more than 72 h were excluded from the study. Severe sepsis and septic shock were diagnosed according to the American–European Consensus Conference (AECC) criteria [21] and managed according to the Surviving Sepsis Campaign guidelines 2012, prevalent during the study period [16].

### Study size

Convenience sample size.

### Data sources/measurement

Sample collection and processing: all patients had invasive arterial pressure monitoring as the standard of care. Blood samples (3 mL each) were collected in ethylene-diamine-tetra-acetic-acid (EDTA) tubes from an arterial catheter at 12 time points: 0 (baseline, before starting infusion), at 5, 15, 30, 60, 90, 120, 180, 240, 300, 360 and 480 min after the first dose. A similar set of 12 samples was repeated on day three (seventh dose) of the regimen. Samples were immediately transferred to an icebox, centrifuged at 4000*g* at 4 °C for 10 min and the supernatant plasma was stored at − 80 °C for subsequent analysis.

#### Meropenem assay

Plasma meropenem concentration was determined using a validated reverse-phase high-performance liquid chromatography (HPLC) assay. The meropenem extraction procedure followed for the study was customized based on the principles of the standard extraction procedures [22, 23]. An aliquot of the extracted sample (30 µL) was injected using an automated injection system (Dionex Autosampler; ThermoFisher) onto a C18 column. The mobile phase consisted of 15 mM KH_2_PO_4_–acetonitrile–methanol (84:12:4, v/v/v), pH 2.8, at a flow rate of 1 mL/min. The column effluent was monitored by a photodiode array (PDA) detector (Dionex; ThermoFisher) at 308 nm. Peaks were recorded and integrated using Chameleon software (Dionex; ThermoFisher) [22, 23]. The limit of quantitation (LOQ) of the assay was 0.1 µg/mL.

### Quantitative variables

Pharmacokinetic modeling and simulation: Pharmacokinetics compartmental analysis and simulation were performed using Phoenix, WinNonlin classic PK modeling software (Certara USA, Inc., NJ). A one-compartment model with zero-order input and first-order elimination was fitted into meropenem plasma profiles, using the least squares method. Randomness of the residuals was assessed visually. Pharmacokinetic parameters including maximum plasma concentration (Cmax), area under concentration–time curve (AUC)—a measure of total drug exposure, elimination half-life (*T*_1/2_), elimination rate constant (Ke), apparent volume of distribution (Vd) and total body clearance (Cl) were estimated for each patient. Further, using the initial estimates from this fitting, the data were simulated to predict drug plasma concentrations at doses ranging from 1500 to 3000 mg administered as a 3-h infusion every eighth hourly (Q8H) or every sixth hourly (Q6H) in the context of bolus doses ranging from 500 to 1500 mg prior to the first dose.

The European Committee on Antimicrobial Susceptibility Testing (EUCAST) breakpoints for the minimum inhibitory concentration (MIC) were used. Current breakpoints for susceptible and resistant strains of Enterobacteriaceae, *Pseudomonas aeruginosa* (PsA) and *Acinetobacter baumannii* (AcB) are ≤ 2 µg/mL and > 8 µg/mL, respectively.

### Study endpoints

The primary endpoint was the proportion of patients in whom Cp was greater than the breakpoint for sensitive strains of both Enterobacteriaceae as well as PsA and AcB, for ≥ 40% of the dosing interval (fT > 2 µg > 40). Secondary endpoints were fT > 4 µg/mL > 40, (required for activity against strains susceptible to increased antibiotic exposure), fT > 8 µg/mL > 40 (four times the MIC breakpoint for sensitive strains), fT > 4 µg/mL = 100 and fT > 8 µg/mL = 100. We also looked at the proportion of patients in whom Cp > MIC was achieved within 1 h after the commencement of the first dose.

### Statistical methods

Patient characteristics were analyzed using appropriate descriptive statistics such as mean ± SD or percentages. Pharmacokinetic variables were compared between day 1 and day 3 using the paired *t*-test. In addition, changes in pharmacokinetic variables on day 1 and day 3 between survivors and non-survivors were compared using unpaired *T*-test. *P* < 0.05 was considered statistically significant.

## Results

### Participants

Twenty-five critically ill patients with severe sepsis or septic shock were enrolled in the study from June 2013 to Oct 2014. One patient died before day 3 and another patient had to be withdrawn from the study as the blood samples were severely hemolyzed and hence not suitable for analysis. Thus, 24 sample sets of day 1 and 23 sets of day 3 were available for final analysis.

### Descriptive data

The baseline characteristics and outcomes of these patients are shown in Table 1.

**Table 1:** Patient characteristics

Parameters	Value
Age (years) median (range)	54 (25–70)
Gender	
Male	12
Female	13
APACHE score (mean ± SD)	15.4 ± 8.09
Mean SOFA score (mean ± SD)	
Day 1	7.35 ± 3.62
Day 3	6.07 ± 2.09
Baseline albumin level (g/dL)	2.35 ± 0.8
Baseline serum creatinine (mg/dL)^a^	
Median	0.9
Range	0.5–2.6
Baseline creatinine clearance (mL/min) (mean ± SD)	73.8 ± 26.6
Requirement of mechanical ventilation (*n*, %)	22 (88%)
Requirement of inotropes (*n*, %)	15 (60%)
Median ICU length of stay (IQR)	8 days (5–14)
ICU mortality, n (%)	10 (40%)
Hospital mortality, n (%)	11 (44%)

*APACHE II* Acute Physiology and Chronic Health Evaluation II, *ICU* intensive care unit, *IQR* inter-quartile range, *SD* standard deviation, *SOFA Score*: Sequential Organ Failure Assessment Score

^a^Predicted creatinine clearance calculated based on formula discovered by Cockcroft and Gault

### Endpoints

The 3-h EI of 1000 mg meropenem Q8H achieved fT > 2 µg/mL > 40 in all patients on day 1 and day 3. However, it achieved fT > 4 µg/mL > 40 in 16 out of 24 (66.7%) patients on the first day and 14 out of 23 (60.86%) patients only on the third day (Table 2). The targets of Cp > 2 µg/mL and 4 µg/mL at 1 h following the first dose were achieved in 87.5 and 75% patients, respectively. Less than half the number of patients could achieve fT > 8 µg/mL > 40. None of the patients could achieve fT > 2 µg/mL = 100 and subsequent higher targets of fT > 4 µg/mL = 100 or fT > 8 µg/mL = 100.

**Table 2:** Targets achieved with a 3-h extended infusion of meropenem 1000 mg 8 hourly

End point	Day 1 (Dose 1) (*n* = 24^a^) (%)	Day 3 (Dose 7) (*n* = 23^b^) (%)
Number of patients (%) with fT > 2 μg/mL > 40	24 (100)	23 (100)
Number of patients (%) with fT > 4 μg/mL > 40	16 (66.7)	14 (60.86)
Number of patients (%) with fT > 8 μg/mL > 40	9 (37.5)	8 (34.7)
Number of patients (%) with fT > 4 μg/mL > 100	0 (0)	0 (0)
Number of patients (%) with fT > 8 μg/mL > 100	0 (0)	0 (0)
Number of patients (%) achieving Cp > 2 μg/mL within 1 h	21 (87.5)	–
Number of patients (%) achieving Cp > 4 μg/mL within 1 h	18 (75)	–

fT > 2 μg/mL > 40: Plasma meropenem concentration exceeds 2 μg/mL for more than 40% of the 8-h dosing interval. fT > 4 μg/mL > 40: Plasma meropenem concentration exceeds, 4 μg/mL for more than 40% of the 8-h dosing interval. Cp > 2 μg/ml: Plasma meropenem concentration more than 2 μg/mL. Cp > 4 μg/mL: Plasma meropenem concentration more than 4 μg/mL

^a^One patient was withdrawn from the analysis as the blood samples were hemolyzed. ^b^One patient expired before collection of day 3 samples

### Pharmacokinetic analysis

Table 3 shows the pharmacokinetic parameters following the first dose and after the seventh dose on day 3, representing the steady state. No significant difference in maximum plasma concentration (Cmax) or total exposure to meropenem (AUC) was observed between the 2 days. The volume of distribution and clearance on day 1 and day 3 are shown for survivors, non-survivors and all patients in (Additional file 1: Fig. S1). The difference between day 1 and day 3 was not statistically significant for either parameter in any of the groups. There was a marked but statistically non-significant decrease in Vd (39.2%) and increase in Cl (32.4%) from day 1 to day 3. This change (Δ) from day 1 to day 3 was more pronounced in survivors compared to non-survivors (ΔVd 44.46% vs 25.30% and ΔCl 44.41% vs 15.55%); however, the difference was not statistically significant.

**Table 3:** Pharmacokinetic parameters after 3-h extended infusion (EI) of 1000 mg meropenem 8 h for first and third days

Pharmacokinetic parameters	Day 1 (first dose) (*n* = 24^a^)	Day 3 (seventh dose) (*n* = 23^b^)	Change^c^ from day 1 to day 3 (%)	*P*^d^
Cmax (μg/mL)	15.36 ± 1.11	14.14 ± 2.02	− 7.1	NS
AUC (μg h/mL)	57.92 ± 5.98	43.82 ± 7.33	− 24.3	NS
T_1/2_ (h)	1.31 ± 0.24	0.6 ± 0.23	− 54.2	0.04
Ke (1/h)	0.53 ± 0.10	1.15 ± 0.44	+ 116.1	NS
Vd (L)	32.61 ± 4.3	19.83 ± 6.13	− 39.2	NS
Cl (L/h)	17.26 ± 1.78	22.86 ± 3.82	+ 32.4	NS

All values shown as mean ± SE

*Cmax* maximum plasma concentration, *NS* not significant, *AUC* area under concentration–time curve, *T*_1/2_ half-life, *Ke* elimination rate constant, *Vd* apparent volume of distribution, *Cl* total body clearance

*P* < 0.05 statistically significant

^a^One patient was withdrawn from the analysis as the blood samples were hemolyzed

^b^One patient expired before collection of day three samples

^**c**^(+) indicates increase and (−) indicates decrease from day 1 to day 3

^d^Paired data of 23 patients between day 1 and day 3 compared using paired *t*-test

### Pharmacokinetics modeling and simulation

**Fig. 1:**
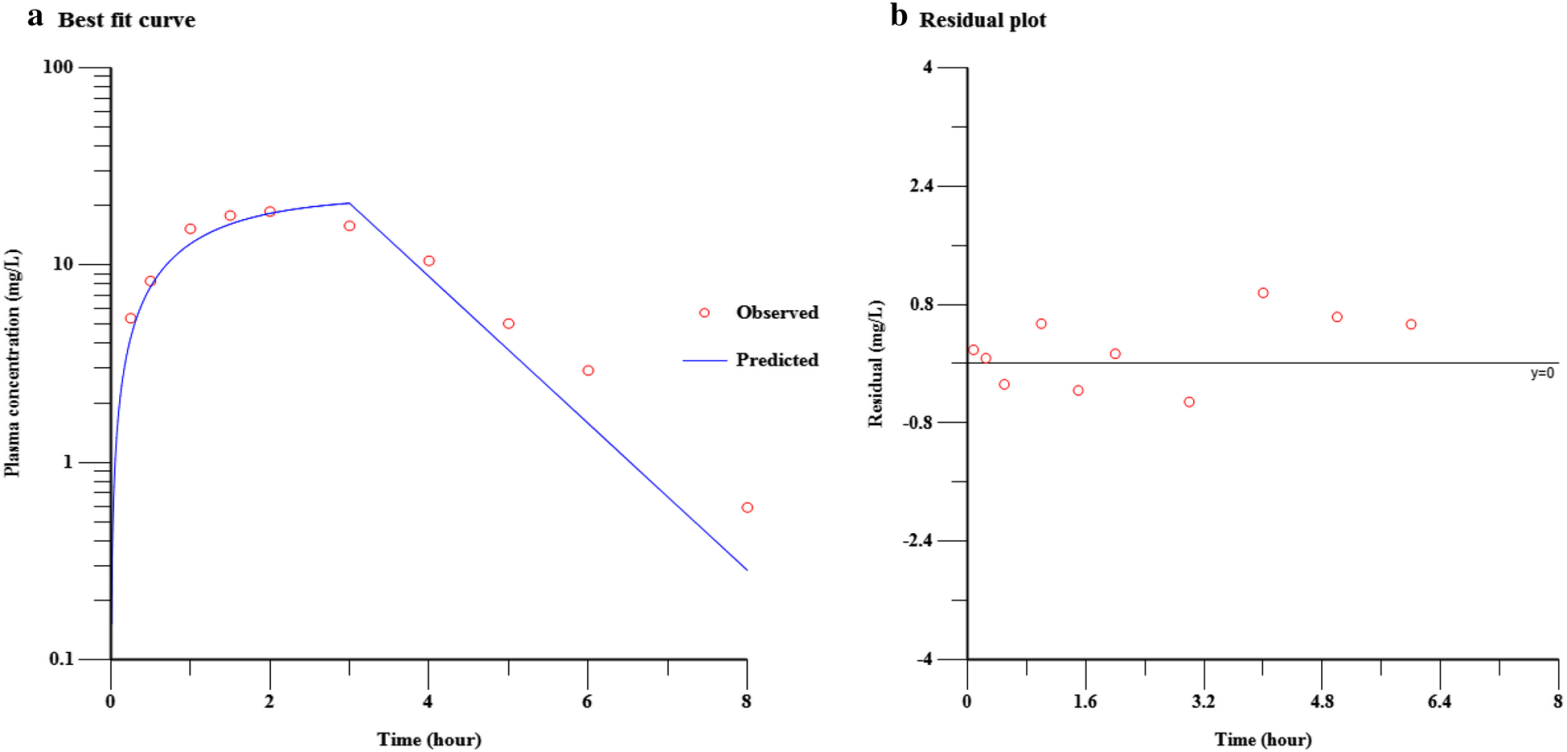
The best fit curve (**a**) and the residual plot (**b**) of a representative patient showing one-compartment PK fitting of meropenem. **a** Open red dots represent meropenem concentrations at different sampling times. Curve fitting is a mathematical function that has the best fit to a series of data points. **b** Random distribution of residuals, i.e., difference between the observed concentration and that predicted by the model on the residual plot indicates that the model is appropriate for the data

Meropenem plasma kinetics was found to fit a one-compartment model when given as a 3-h infusion at the dose of 1000 mg. A representative fit and residual plot of the first dose kinetics are shown in Fig. 1. Using the initial estimates from this fitting, the data were simulated to predict drug plasma concentrations at various doses between 1500 and 3000 mg administered as a 3-h infusion at Q8H and 6 hourly (Q6H) with the preceding bolus dose of 500–1500 mg (Additional file 2: Fig. S2. The proportion of patients, achieving the pharmacokinetic targets in each of these situations based on simulation is shown in Table 4 and Additional file 3: Fig. S3 and Additional file 4: Fig. S4. Clearly, administration of a bolus of 500 mg prior to the first dose could have resulted in achieving the target exposure of fT > 4 µg/mL > 40 in all patients when followed by 1500 mg of meropenem as a 3-h EI Q8H. However, this regimen was not efficient for prolonged targets like fT > 4 µg/mL = 100 and fT > 8 µg/mL = 100 due to the rapid decline of meropenem concentration between 6 and 8 h. These targets require an increase in both the dose and the frequency of administration from Q8H to Q6H to achieve these targets in the majority of the patients (Additional file 4: Fig. S4).

**Table 4:** Simulation results on the first and third days showing the percentage of patients achieving target exposures for different doses administered as 3-h extended infusion (EI) after 500-mg IV bolus prior to first dose on day 1

Target	fT > 4 μg/mL > 40		fT > 4 μg/mL = 100	
Regimen with 500 mg IV bolus prior to first dose on day 1	Day 1 (*N *= 24)	Day 3 (*N *= 23)	Day 1 (*N *= 24)	Day 3 (*N *= 23)
1500 mg 8 hourly	24 (100%)	23 (100%)	09 (37.5%)	11 (48%)
2000 mg 8 hourly	24 (100%)	23 (100%)	09 (37.5%)	14 (61%)
1500 mg 6 hourly	24 (100%)	23 (100%)	15 (63%)	20 (87%)
2000 mg 6 hourly	24 (100%)	23 (100%)	16 (67%)	23 (100%)

fT > 4 μg/mL > X: plasma meropenem concentration exceeds 4 μg/mL for more than X% of the dosing interval

## Discussion

Our study shows that giving 1000 mg meropenem as a 3-h EI Q8H can effectively achieve the target (fT > 2 µg/mL > 40%), providing adequate activity only against susceptible strains of Enterobacteriaceae, PsA and AcB. However, this regimen does not achieve fT > 4 µg/mL > 40% in one-third of patients. Also, Cp > 4 μg/mL within 1 h of starting the first dose is not achieved in one-fourth of patients with meropenem EI without a preceding bolus. These findings suggest that the routinely used EI of 1000 mg meropenem may not be adequate for empiric coverage of all non-resistant strains of these Gram-negative bacteria which are susceptible to increased exposure. The PK modeling and simulation showed that a dose of 1500 mg meropenem as a 3-h EI Q8H with a preceding bolus of 500 mg before the first dose would achieve the pharmacokinetic target of fT > 4 µg/mL > 40% in most of the patients.

Pharmacokinetics of meropenem are likely to be altered in patients with severe sepsis compared to healthy volunteers because of infusion of a large volume of fluids and blood products, increased capillary permeability, high cardiac output, augmented renal clearance, or renal hypoperfusion [5]. Therefore, not surprisingly, Vd was found to be higher in our patients than reported in healthy individuals [24]. The implications of our findings are that patients with severe sepsis or septic shock would require higher doses to account for increased Vd. Studies in which an EI was preceded by a bolus of meropenem have shown that the PK goals were achieved in a majority of patients [12, 17, 18]. A bolus dose preceding the EI would increase the Cmax as well as the Cp in the first hour. However, several other studies have shown that an EI without a preceding bolus is equally effective in conditions such as ventilator-associated pneumonia, febrile neutropenia with bacteraemia, hematopoietic stem cell transplantation, suspected Gram-negative infections and critically ill patients with septic shock requiring continuous renal replacement therapy [10, 13, 24–29]. In most of these studies, Cp remained greater than MIC for a greater fraction of time with the EI of meropenem without a prior bolus, compared to a 1-h infusion administration of meropenem. Our current regimen was based on these studies. However, we observed delayed Cp > 4 μg/mL in our patients. Based on our pharmacokinetic modeling, we determined that a bolus of 500 mg of meropenem would achieve Cp of 4 μg/mL and a bolus of 1500 mg would be sufficient to achieve Cp of 8 μg/mL almost immediately.

EI of 1000 mg of meropenem could not achieve the pharmacodynamic goal of fT > 4 µg/mL > 40%. Since meropenem is usually initiated in ICU as empirical therapy, it may be highly desirable for the dose to cover most of the organisms including the strains with MIC breakpoints higher than 2 µg/mL which can be susceptible at an increased exposure. Based on our pharmacokinetic modeling, meropenem at a dose of 1500 mg, rather than 1000 mg, given as an EI with a preceding bolus of 500 mg before the first dose would achieve this therapeutic target. Many authors believe in a more aggressive pharmacodynamic target of fT > MIC = 100% for prolonged or continuous infusions [30]. However, meropenem is known to have post-antibiotic effect (PAE) against both Gram-positive and Gram-negative organisms [31], particularly when Gram-positive and Gram-negative organisms like *E. coli* and PsA were exposed to the meropenem levels four times the MIC for 2 h [32].

In our PK modeling and simulation, a target of fT > 4 µg/mL = 100 could be achieved in very few patients with Q8H dosing. Hanberg et al. have also observed attainment of fT > MIC > 40 and inability of the meropenem EI to achieve both fT > MIC = 100, and fT > 4 × MIC = 100 [33]. We observed a rapid fall of Cp between 6 and 8 h and the greater probability of achieving fT > 4 µg/mL = 100 and fT > 8 µg/mL = 100 by increasing the frequency to Q6H instead of Q8H. A follow-up study with this regimen would validate this hypothesis.

The PK characteristics of meropenem in plasma described in the present study are similar to those from previous studies performed in critically ill patients [10–12]. Patients with sepsis and septic shock are known to have variable pharmacokinetics. The decrease in Vd (39.2%) and an increase in Cl (32.4%) from day 1 to day 3 in our study was not statistically significant. Further, no statistically significant difference was observed between survivors and non-survivors with respect to changes in Vd and Cl from day 1 to day 3.

We could look at the shortcomings of the existing practice of meropenem EI with no preceding bolus since we collected the blood sample at frequent intervals during the first hour of the first dose. Taccone et al. studied a 30-min meropenem 1000 mg infusion over Q8H preceded by a bolus of 1000 mg and looked at the meropenem drug levels at multiple time points including 1 h. They targeted the drug level of 8 μg/mL which is four times the MIC breakpoint for sensitive Gram-negative organisms and fT > 40% as a target for meropenem. The target of fT > 8 µg/mL > 40% was achieved in only 57% of the patients [34].

A feature of our study is the inclusion of the data both for the initial dose on day 1 as well as the seventh dose on day 3, allowing us to capture the changing pharmacokinetic profile of the drug. The observation of a decrease in Vd and an increase in Cl from the first day to the third day in our study may be studied further. This may help in optimization of the doses.

Other strengths of our study include the relatively large sample size for the evaluation of pharmacokinetics of extended infusion of meropenem, and the inclusion of patients with severe sepsis or septic shock, with mean Acute Physiology and Chronic Health Evaluation II (APACHE II) score of 15.4 and Sequential Organ Failure Assessment Score (SOFA) score of 8.6 with a majority of patients receiving mechanical ventilation and vasopressors. The pharmacokinetics in these patients are likely to be significantly altered. We enrolled patients based on the prevailing definition of sepsis at the time of the study, rather than the current Sepsis-3 criteria [35].

We could harness the power of modeling and simulation to identify the right strategy for dosing meropenem. Modeling and simulation are being increasingly used in modern-day medicine for drug development as well as PK-driven optimization of drugs. An obvious strength of simulation lies in the fact that various scenarios can be simulated using a few patients’ data, which could be subsequently validated in a small cohort of patients, thus obviating the need for large dose-ranging studies. This enables optimal therapeutic strategies to be adopted faster in clinical practice. The currently recommended high dose of meropenem (2000 mg Q8H) can achieve fT > 8 µg/mL > 40 in most of the patients with an EI along with a 500 mg preceding bolus (Supplemental Digital Content—Table 1).

It is pertinent to mention here that rather than using a population pharmacokinetics (popPK) analysis, we resorted to compartment modeling for the estimation of Vd and Cl for each patient from which PK profiles were simulated for various dosing scenarios. Several authors in the past have used the popPK approach to analyze such data [24, 29]. While popPK is very informative to identify the sources and correlates of variability, the relatively small sample size would have affected the model estimates. Compartment modeling, on the other hand, allows the estimation of the duration of time for which concentrations were above a predefined threshold which was the primary objective of our study. Since a rich sampling strategy was followed in our study, these estimates are likely to be highly accurate. Two recent PopPK studies of meropenem in ICU patients have demonstrated one-compartment pharmacokinetics with first-order elimination which corroborates with the similar pharmacokinetic profile observed in our study [36, 37].

One of the limitations of our study is the exclusion of potentially very sick patients with calculated creatinine clearance < 50 mL/min and those not expected to survive for 72 h. This was necessary, as we planned to look at steady-state levels on the third day. We have not analyzed whether the difference in meropenem exposure has any effect on mortality. Our study was not designed to answer this question.

## Conclusions

In patients with severe sepsis or septic shock, a 3-h EI of meropenem 1000 mg Q8H achieved fT > 2 µg/mL > 40 both on the first and third days, providing adequate coverage against sensitive strains of Enterobacteriaceae, PsA and AcB. However, it failed to achieve fT > 4 µg/mL > 40 for activity against non-resistant strains of these organisms susceptible to increased exposure in 33.3 and 39.1% patients on the first day and the third day, respectively. A bolus of 500 mg followed by EI of 1500 mg Q8H can achieve this target in all patients. Higher doses and increasing dose frequency are required for the PK targets fT > 8 µg/mL > 40, fT > 4 µg/mL = 100 and fT > 8 µg/mL = 100.

## Supplementary information

**Additional file 1: Fig. S1.** Volume of distribution (A) and clearance (B) on day 1 and day 3 are shown for survivors, non-survivors and all patients. The difference between day 1 and day 3 was not statistically significant for either parameter in any of the groups.

**Additional file 2: Fig. S2.** Simulation of individual patient’s concentration–time profile for three hour extended infusion of 1500 mg, 2000 mg, 2500 mg and 3000 mg dose of meropenem following bolus doses of 500 mg (a–d), 1000 mg (e–h) and 1500 mg (i–l), respectively, is shown. The dotted lines at 2, 4, 8 and 16 µg/mL represent various MIC thresholds. The vertical lines to the right at 6 hour and 8 hour are shown to indicate dosing frequencies. It s clear from these simulations that longer exposures over MIC (ft > MIC = 100) can be achieved only by increasing the frequency of dosing from eight hourly (Q8H) to six hourly (Q6H). MIC: Minimum Inhibitory Concentration.

**Additional file 3: Fig. S3.** Results of simulation (N = 24 patients) showing the number of patients achieving the therapeutic target of fT > MIC > 40 at minimum inhibitory concentration (MIC) ranging from 2 to 16 µg/mL for various bolus doses viz. 500 mg, 1000 mg and 1500 mg of meropenem. Infusion doses ranging from 1500 mg to 3000 mg administered over 3 hours at eight hourly intervals were used for simulation. The European Committee on Antimicrobial Susceptibility Testing (EUCAST) defines MIC of < 2 µg/ml as ‘sensitive’ and > 8 µg/ml as ‘resistant’ for Enterobacteriaceae, Pseudomonas aeruginosa (PsA) and Acinetobacter baumannii (AcB). Non-resistant strains with MIC > 2 µg/ml can be susceptible to increased exposure.

**Additional file 4: Fig. S4.** Results of simulation (N = 24 patients) showing the number of patients achieving the therapeutic target of fT > MIC > 100 at minimum inhibitory concentration (MIC) ranging from 2–16 µg/mL for various bolus doses viz. 500 mg (A), 1000 mg (B) and 1500 mg (C) of meropenem. Infusion doses ranging from 1500 mg to 3000 mg administered over 3 h at eight hourly (Q8H) and six hourly (Q6H) intervals were used for simulation. The European Committee on Antimicrobial Susceptibility Testing (EUCAST) defines MIC of < 2 µg/ml as ‘sensitive’ and > 8 µg/ml as ‘resistant’ for Enterobacteriaceae, Pseudomonas aeruginosa (PsA) and Acinetobacter baumannii (AcB). Non-resistant strains with MIC > 2 µg/ml can be susceptible to increased exposure.

## Data Availability

The datasets used and/or analyzed during the current study are available from the corresponding author on reasonable request

## Abbreviations

Cp: plasma concentration
MIC: minimum inhibitory concentration
fT > MIC > 40: plasma concentration (Cp) above the minimum inhibitory concentration (MIC) of the pathogen for at least 40% of dosing interval
EI: extended infusion
Q8H: every 8 h/eight hourly
Q6H: every 6 h/six hourly
PK: pharmacokinetic
PD: pharmacodynamic
ICU: intensive care unit
Cmax: peak plasma concentration
AUC: area under concentration–time curve
*T*_1/2_: elimination half-life
Ke: elimination rate constant
Vd: apparent volume of distribution
Cl: total body clearance
GNB: Gram-negative bacteria
