# Supplementary figures and images for: Clinical pharmacokinetics of 3-h extended infusion of meropenem in adult patients with severe sepsis and septic shock: implications for empirical therapy against Gram-negative bacteria
Source: Ann Intensive Care. 2020 Jan 10;10:4. doi: 10.1186/s13613-019-0622-8 (PMC6954163; doi:10.1186/s13613-019-0622-8)

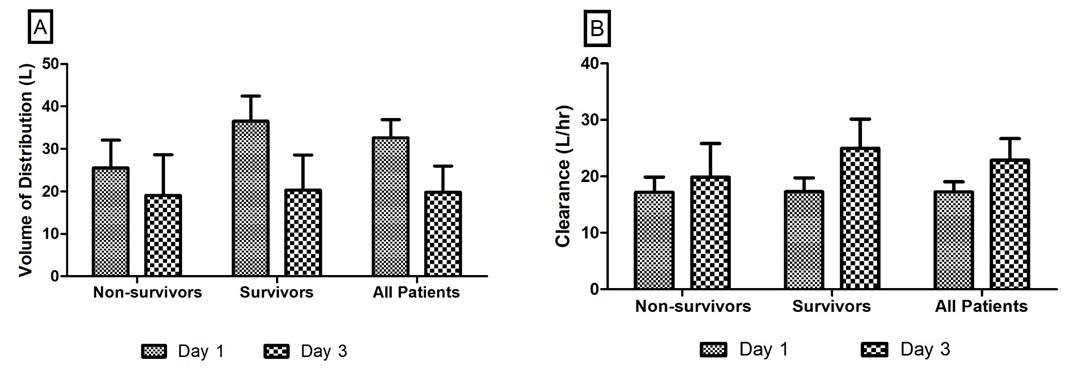

Supplement: Supplementary file 1 — Additional file 1: Fig. S1. Volume of distribution (A) and clearance (B) on day 1 and day 3 are shown for survivors, non-survivors and all patients. The difference between day 1 and day 3 was not statistically significant for either parameter in any of the groups. [file 13613_2019_622_MOESM1_ESM.jpg]

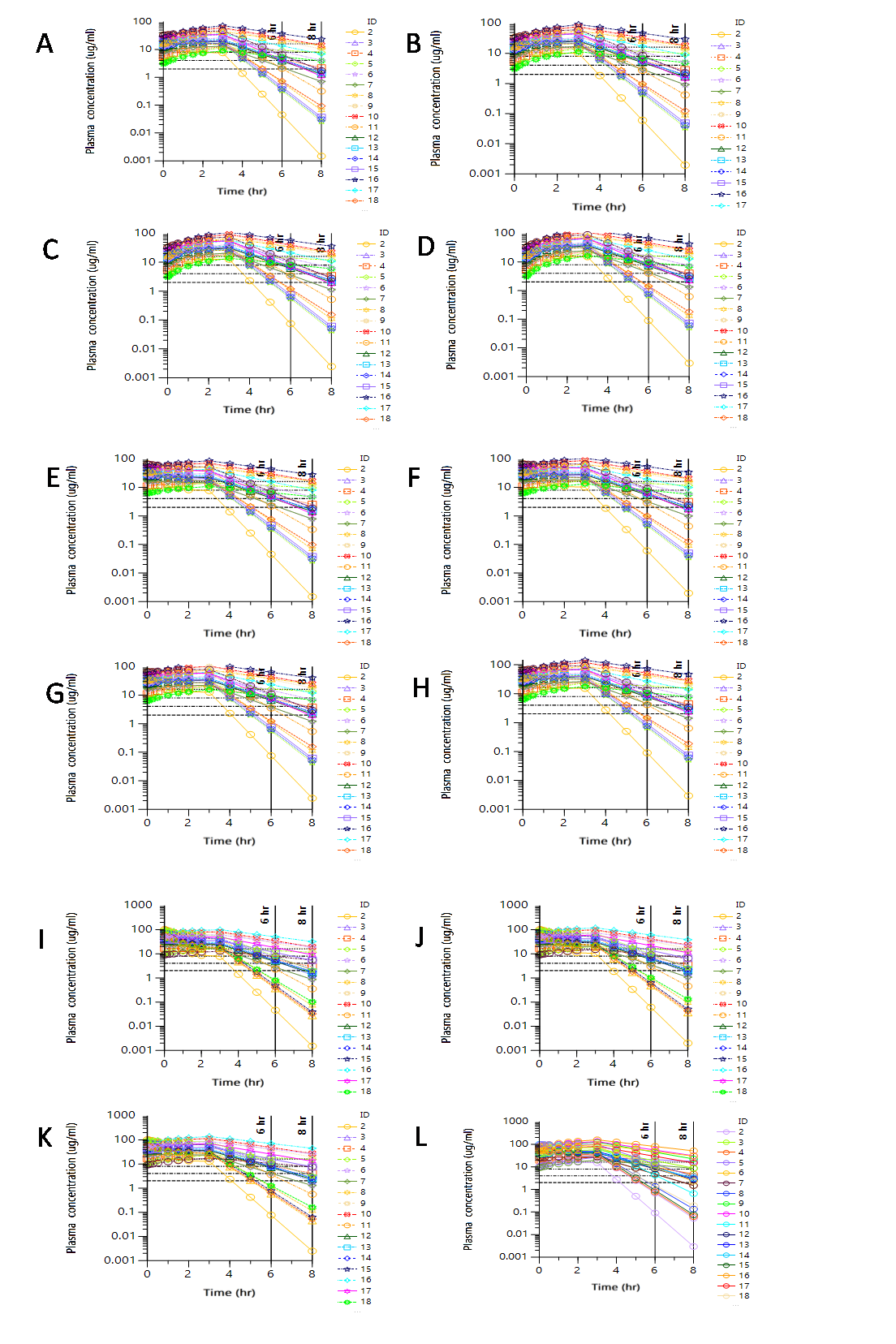

Supplement: Supplementary file 2 — Additional file 2: Fig. S2. Simulation of individual patient’s concentration–time profile for three hour extended infusion of 1500 mg, 2000 mg, 2500 mg and 3000 mg dose of meropenem following bolus doses of 500 mg (a–d), 1000 mg (e–h) and 1500 mg (i–l), respectively, is shown. The dotted lines at 2, 4, 8 and 16 µg/mL represent various MIC thresholds. The vertical lines to the right at 6 hour and 8 hour are shown to indicate dosing frequencies. It s clear from these simulations that longer exposures over MIC (ft > MIC = 100) can be achieved only by increasing the frequency of dosing from eight hourly (Q8H) to six hourly (Q6H). MIC: Minimum Inhibitory Concentration. [file 13613_2019_622_MOESM2_ESM.tif]

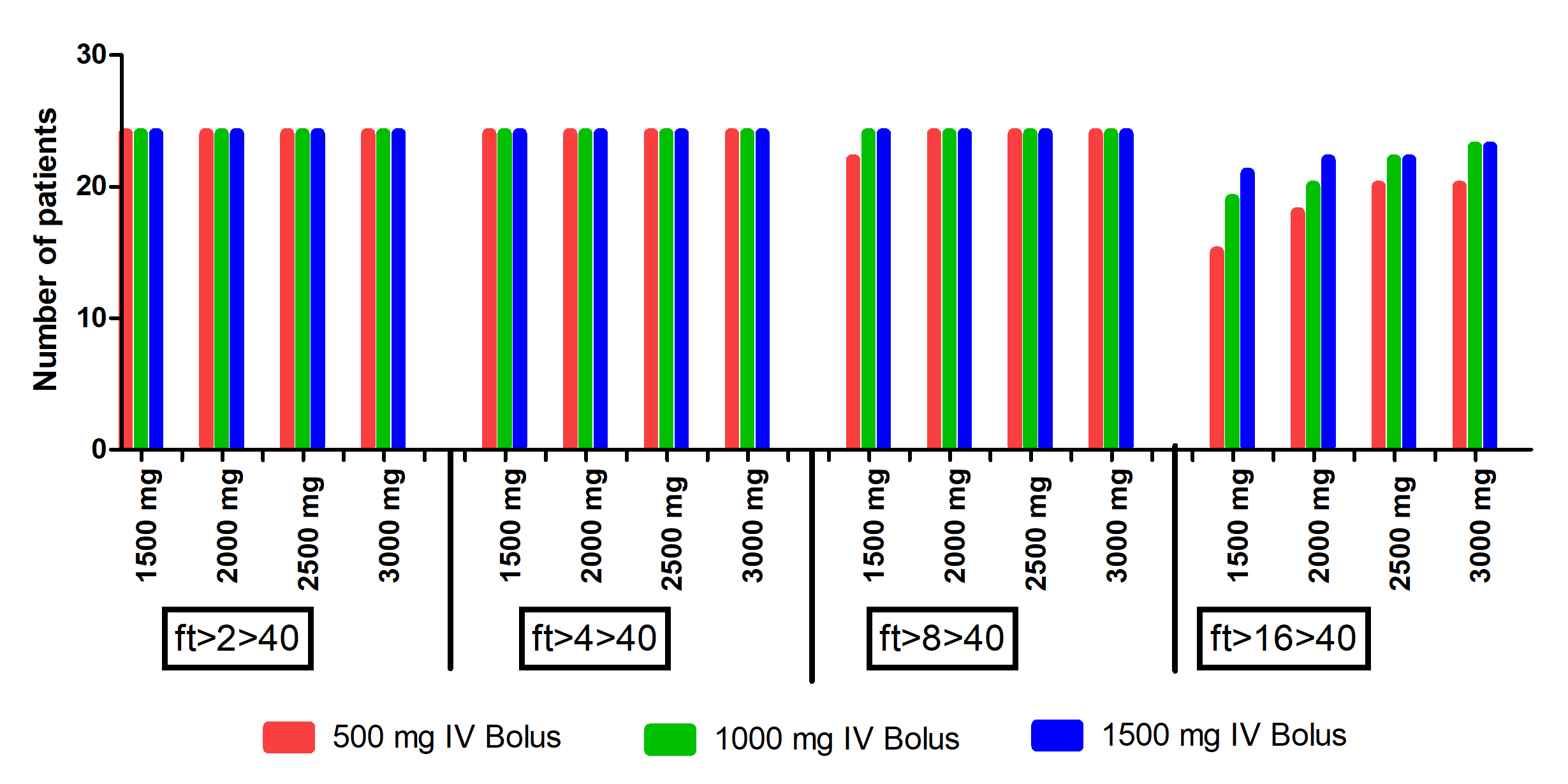

Supplement: Supplementary file 3 — Additional file 3: Fig. S3. Results of simulation (N = 24 patients) showing the number of patients achieving the therapeutic target of fT > MIC > 40 at minimum inhibitory concentration (MIC) ranging from 2 to 16 µg/mL for various bolus doses viz. 500 mg, 1000 mg and 1500 mg of meropenem. Infusion doses ranging from 1500 mg to 3000 mg administered over 3 hours at eight hourly intervals were used for simulation. The European Committee on Antimicrobial Susceptibility Testing (EUCAST) defines MIC of < 2 µg/ml as ‘sensitive’ and > 8 µg/ml as ‘resistant’ for Enterobacteriaceae, Pseudomonas aeruginosa (PsA) and Acinetobacter baumannii (AcB). Non-resistant strains with MIC > 2 µg/ml can be susceptible to increased exposure. [file 13613_2019_622_MOESM3_ESM.jpg]

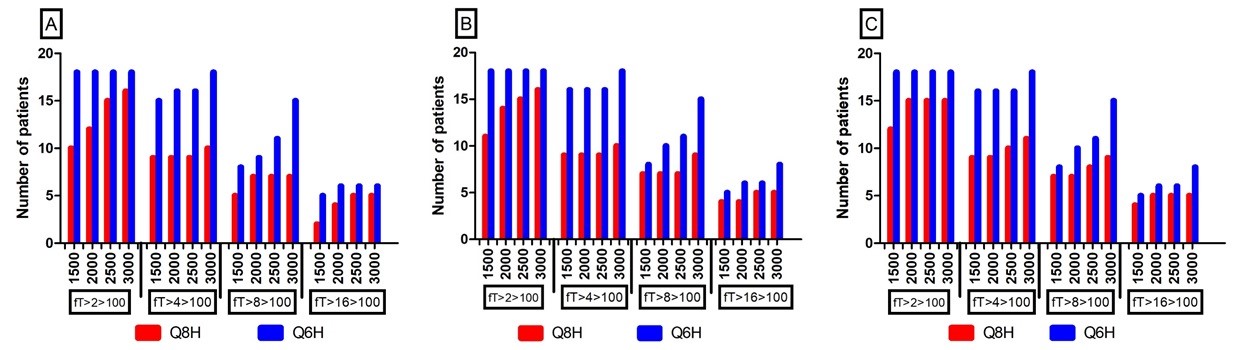

Supplement: Supplementary file 4 — Additional file 4: Fig. S4. Results of simulation (N = 24 patients) showing the number of patients achieving the therapeutic target of fT > MIC > 100 at minimum inhibitory concentration (MIC) ranging from 2–16 µg/mL for various bolus doses viz. 500 mg (A), 1000 mg (B) and 1500 mg (C) of meropenem. Infusion doses ranging from 1500 mg to 3000 mg administered over 3 h at eight hourly (Q8H) and six hourly (Q6H) intervals were used for simulation. The European Committee on Antimicrobial Susceptibility Testing (EUCAST) defines MIC of < 2 µg/ml as ‘sensitive’ and > 8 µg/ml as ‘resistant’ for Enterobacteriaceae, Pseudomonas aeruginosa (PsA) and Acinetobacter baumannii (AcB). Non-resistant strains with MIC > 2 µg/ml can be susceptible to increased exposure. [file 13613_2019_622_MOESM4_ESM.jpg]
